# Transpulmonary pressure during high-frequency oscillation ventilation: Is it the culprit?

**DOI:** 10.1186/s13613-016-0191-z

**Published:** 2016-09-13

**Authors:** M. Cressoni, Davide Chiumello

**Affiliations:** 1 Dipartimento di Fisiopatologia Medico Chirurgica e dei Trapianti, Università degli Studi di Milano, Milan, Italy; 2 Dipartimento di Scienze della Salute, Università degli Studi di Milano, Milan, Italy; 3 Dipartimento di Emergenza-Urgenza, ASST Santi Paolo e Carlo, Milan, Italy

High-frequency oscillation ventilation (HFOV) seems the perfect embodiment of the “open lung theory” as it suggests extremely low tidal volumes combined with very high mean airway pressures. With disappointment clinical trials showed that the application of HFOV was associated with a decreased survival [1]. The culprit has not been identified, and a possible cause is the effect of increased intrathoracic pressures on the right ventricle and very high intrathoracic pressures which act as an obstacle to blood flow [1]. When a positive pressure is applied to the respiratory system, it is spent in part to inflate the lung and in part to inflate the chest wall. The real distending force of the lung is the transpulmonary pressure (TP) which is the difference in pressure between the pleural space and the alveolar units. As pleural pressure cannot be directly measured, the esophageal pressure is used as a surrogate.

In a recent article published in Annals of Intensive Care, Guervilly et al. [2] explored in a small ARDS patient population (10 patients) the transpulmonary pressure during HFOV and conventional mechanical ventilation (CMV) to compare the range of TP occurring during the switch from CMV to an HFOV trial. The authors performed three steps of HFOV with mean airway pressure levels 5, 15 and 15 cmH_2_O greater than the CMV resulting in 23 ± 4, 28 ± 4 and 33 ± 4 cmH_2_O, respectively. The absolute level of esophageal pressure increased with the increase in airway pressure (12 [10–17], 17 [13–19] and 19 [17–23] cmH_2_O). An increase in the absolute level of esophageal pressure is a common observation when airway pressure is increased, as an example increasing positive end-expiratory pressure (PEEP) [3]. The increase in absolute esophageal pressure is function of the airway pressure increase and of the ratio between chest wall elastance and respiratory system elastance according to the following equation:

$${\text{Expected}}\;{\text{change}}\;{\text{in}}\;{\text{absolute}}\;{\text{esophageal}}\;{\text{pressure}}\left( {{\text{cmH}}_{2} {\text{O}}} \right) = \Delta \;{\text{Airway}}\;{\text{pressure}}\left( {{\text{cmH}}_{2} {\text{O}}} \right)\,*\,{\text{Chest}}\;{\text{wall}}\;{\text{elastance/Respiratory}}\;{\text{system}}\;{\text{elastance }}\left( {{\text{cmH}}_{2} {\text{O}}/{\text{L}}} \right)$$

where ∆ Airway pressure (cmH_2_O) is the change in airway pressures (cmH_2_O) between the two PEEP levels [3].

It follows that if esophageal pressure would not increase while increasing airway pressure, the chest wall elastance is bounded to increase and all the pressure increase would spend in inflating the chest wall and not the lung.

We can model the three HFOV steps of the study as three inspirations starting from zero end-expiratory pressure (ZEEP) and going to the average airway pressure applied during HFOV. In classical physiological literature, it is usually accepted that the changes in esophageal pressure correspond to the changes in pleural pressure. Indeed, some authors suggest that the absolute value of esophageal pressure corresponds to the real pleural pressure in the mid-lung [4]. This possibility is supported by scarce physiological data [5] as direct measurement of pleural pressure is challenging. If we accept these assumptions, TP was similar between HFOV +5 (10.5 [7.3;13.8] cmH_2_O) and CMV (8 [6;13] pre-study and 12 [5;12] post-study), while it was slightly greater at HFOV +10 and +15 (13 [9;15] and 14 [12;16]). An alternative approach, if one does not believe in the absolute esophageal pressure, is to compute the TP from the differences in airway and esophageal pressures, which are known to approximate the changes in esophageal pressure [3]. In this case, the delta TP would have been 12, 14 and 17 cmH_2_O.

Anyway, the absolute TP values, whatsoever computed, are few cmH_2_O higher than during CMV and similar to the ones published at 15 cmH_2_O PEEP in ARDS patients during CMV. Are these data sufficient to infer that during HFOV the possible ventilator-induced lung injury (VILI) is associated with overinflation? We do not think so. It may be objected that during HFOV the higher TP is applied during the whole respiratory cycle and not only during inspiration and so the average stress applied to the lung is greater. This question may be broadly re-formulated asking which component of the inspiratory cycle is better associated with VILI? Plateau pressure? Average airway pressure? As the mechanical ventilator performs a mechanical work on the lung parenchyma, all the ventilator-related variables can be unified if the mechanical work is measured: at each breath energy is transmitted by the ventilator to the lungs and, if excessive, may disrupt chemical bounds and trigger inflammation [6]. During HFOV, very small tidal volumes are delivered with a very high-frequency summing to a possible elevated mechanical power [1, 6]. In physics, the definition of mechanical work is pressure (absolute value) times delta volume and consequently includes the effect of both the tidal volume and the elevated TP. The mechanical power concept may conciliate the dismal results of HFOV clinical trials with the evidence that VILI depends mostly on the dynamic component of mechanical ventilation. Precise measurements of the mechanical power delivered during HFOV are needed to test this hypothesis, as it is believed that HFOV performs a sort of “massage” on the lung, but the exact interaction between the small tidal volumes of HFOV and the lung is not known.
